# Cultural Variations in Public Beliefs about Mental Disorders: A Comparison between Tunisia and Germany

**DOI:** 10.2174/1745017902016010070

**Published:** 2020-07-30

**Authors:** Matthias C. Angermeyer, Mauro G. Carta, Rym Ghachem, Herbert Matschinger, Aurélie Millier, Tarek Refai, Georg Schomerus, Mondher Toumi

**Affiliations:** 1 Center for Public Mental Health, Gösing am Wagram, Austria; 2 Department of Public Health and Clinical and Molecular Medicine, University of Cagliari, Cagliari, Italy; 3 Razi Hospital, La Mannouba, Tunisia; 4 Institute of Social Medicine, Occupational Health and Public Health, University of Leipzig, Leipzig, Germany; 5 Institute of Health Economics and Health Services Research, University of Hamburg, Hamburg, Germany; 6 Aurélie Millier, Creativ-Ceutical, Paris, France; 7 Tarek Refaï, Creativ-Ceutical, Les Berges du Lac, Tunisia; 8 Department of Psychiatry, University of Greifswald, Greifswald, Germany; 9 Department of Public Health, Aix-Marseille University, Marseille, France

**Keywords:** Public beliefs, Mental illness, Tunisia, Population survey, Mental health, Psychological treatment

## Abstract

**Background:**

In recent years there is a growing interest in public beliefs about mental disorders. Numerous representative population-based studies have been conducted around the globe, also in European countries bordering on the Mediterranean Sea. However, relatively little is known about public beliefs in countries in Northern Africa.

**Objective:**

To fill this gap by comparing public beliefs about mental disorders in Tunisia and Germany, focusing on causal beliefs, help-seeking recommendations and treatment preferences.

**Methods:**

Representative national population-based surveys have been conducted in Tunisia in 2012 (N = 811) and in Germany in 2011 (N = 1852), using the same interview mode and the same fully structured interview starting with a vignette depicting a person suffering from either schizophrenia or depression.

**Results:**

In Tunisia, the public was more likely to adopt psychosocial and to reject biogenetic explanations than in Germany. Correspondingly, psychological treatments were more frequently recommended and biological ones more frequently advised against. There was also a strong inclination to share religious beliefs and to recommend seeking religious advice. Tunisians tended much more than Germans to hold moralistic views and to blame the afflicted person for his or her illness. In Tunisia, the public tended less to differentiate between schizophrenia and depression than in Germany.

**Conclusion:**

Marked differences between Tunisia and Germany exist in public beliefs about the causes of mental disorders and their treatment, which correspond to differences in cultural orientations prevailing in these countries. Mental health professionals need to be sensitive to the particular cultural context in which they operate, in order to be able to reach those they intend to care for.

## 1 INTRODUCTION

In recent years there is a growing interest in public beliefs about mental disorders which is motivated mainly by concerns about the gap between the true and treated prevalence of mental disorders. Apart from issues of insufficient provision of services and fear of stigma, lack of mental health literacy has been discussed as a possible reason for this phenomenon. Numerous studies have been carried out around the globe on symptom recognition, causal beliefs, help-seeking recommend-ations and opinions about appropriate treatment. Main findings were that the public draws a clear line between psychotic disorders and depression. There is a strong tendency to attribute the occurrence of mental disorders to psychosocial factors, particularly psychosocial stress, while biogenetic causes are less frequently endorsed [1]. Consequently, psycho-therapy is preferred over pharmacotherapy. While psychiatrists and psychologists/psychotherapists are equally recommended for the treatment of schizophrenia, for depression psycho-logists/psychotherapists are more recommended, at least in Europe and America [2].

From countries bordering on the Mediterranean Sea, several population-based studies on this subject have also been published, for instance from Spain [3-5], France [6-8], Italy [9-12], Greece [13], Turkey [14], Cyprus [3] and Israel [15, 16]. From North Africa originated so far only one single population study which has been conducted in a northern district of Tunisia [17, 18]. Presenting respondents with common, everyday terms that are used to denote mental illness, this study found that the public makes a sharp distinction between ‘insanity’ and ‘depression’. While for ‘insanity’ various explanations were offered, including life events, biological causes and interpersonal troubles, ‘depression’ was almost exclusively attributed to psycho-social causes, in the first place problems in personal relationships. In the eyes of the public, medication and hospitalization were the first choices for the management of ‘insanity’, for the management of ‘depression’ mainly the provision of social support.

In this paper, we will present results from the first national population-based survey in Tunisia. In contrast to the previous study, case vignettes with descriptions of symptoms of either schizophrenia or depression were employed. We used the same interview as in a study that had been conducted in Germany one year before, which allows direct comparison of results from both countries. This will help bring out more clearly cultural specificities in the conceptualization of mental illness. Based on this comparison we will address the following questions:

- Does the public in Tunisia and Germany hold different beliefs about the causes of schizophrenia and depression?
- Does the public recommend different sources of help or hold different opinions about appropriate treatment?

Better understanding of the mental health beliefs prevailing in the respective cultural context may help increase the sensitivity of mental health professionals to the expectations of their patients and improve the acceptance of the services they offer.

## 2 MATERIALS AND METHODS

### 2.1 Samples

Population surveys were conducted in Germany in 2011 (n = 3642) and in Tunisia in 2012 (n = 834). In Germany, a multistage probability sampling was used with (1) sample points, (2) households, and (3) individuals within the target households. Target households within the sample points were determined according to the random route procedure (*i.e.*, a household was selected randomly as a starting point from where a set route through the area was followed); target persons within households were selected using random digits (response rate 64.0%). In Tunisia, a quota sample was used with stratification for sex, age, family status, socio-economic status and region. In Germany, the sample was drawn only from persons holding German citizenship, in Tunisia from those holding Tunisian citizenship. Eligible respondents were non-institutionalized persons, in Germany aged 18 years and over, in Tunisia between 15 and 65 years. The socio-demographic characteristics of both samples are reported in Table **1**. Comparison with official statistical data shows that our samples reflect fairly well the socio-demographic composition of the general population of both countries, except for an underrepresentation of illiterate persons in Tunisia due to methodological reasons (some questions had to be answered using paper and pencil).

The study protocol has been approved by the ethics committee of Universitätsmedizin Greifswald. Informed consent was considered to have been given when individuals agreed to complete the interview. Trained interviewers recruited from the respective countries conducted the interviews. Fieldwork was done by companies specialized in market and social research, in Germany by USUMA GmbH (Berlin), in Tunisia by BJKA Consulting (Tunis). For the sake of comparability of samples, only respondents aged 18 to 65 years were included in this study. We also included only respondents who had been presented with a vignette depicting a case of either schizophrenia or major depression. This resulted in an effective sample size of 931 persons in Germany and 404 persons in Tunisia presented with the schizophrenia vignette, and 921 persons in Germany and 407 persons in Tunisia presented with the depression vignette.

### 2.2 Interview

In both surveys, the same interview mode (face-to-face) was used. The interview had originally been developed in Germany and has already been employed in several surveys. On both occasions, the fully structured interview was identical as concerns wording and sequence of questions. At the beginning of the interview, respondents were presented with a vignette of a diagnostically unlabeled psychiatric case history. Then, respondents were asked a series of questions to assess their beliefs about the disorder described in the vignette as well as their attitudes towards the person afflicted by it. For use in this study, the interview was translated into colloquial Arab by a translator. A bilingual panel reviewed the translation, looking for any inconsistencies between the original and the translated version. The translated interview was then back-translated by an independent translator and both versions were compared by the panel. If significant differences were apparent, they were discussed until equivalence between both versions was achieved.

In Germany, vignettes depicting a case of either schizo-phrenia or major depressive disorder or alcohol dependence were used, in Tunisia only vignettes with schizophrenia and depression. The symptoms described in the vignettes fulfilled the criteria of DSM-IV for the respective disorder. In a previous study, the vignettes had independently been rated by five experts on psychopathology masked to actual diagnosis, providing confirmation of the correct diagnosis for each case history. The sex of the individual presented in the vignettes was randomly varied. Respondents were randomly allocated to receive one of the vignettes.

### 2.3 Measures

In both countries, we elicited beliefs about possible causes of the disorders described in the vignette with a list of 13 possible causes, each of which had to be rated on a five-point Likert scale anchored with 1=‘certainly a cause’ and 5=‘certainly not a cause’. The causes offered represent the following four domains: current stress (stressful life event, work-related stress, problems with partner and family), childhood adversities (grown up in a broken home, lack of parental affection, childhood sexual abuse), biogenetic causes (chemical imbalance in the brain, brain disease and heredity) and moralistic beliefs (immoral life style, weak will). In addition, only in Tunisia three supernatural explanations were offered: no faith in God, no religious commitment, possessed by djinns.

We then asked the respondents to indicate, using a 5-point Likert scale with the anchors “would strongly recommend” and “would not recommend at all", to what extent they would recommend various sources of help. The following options were offered: confidant, health cure, G.P., psychiatrist, psychotherapist, internet, priest or imam, and (only in Tunisia) exorcist/traditional healer.

Finally, respondents were presented with a list of treatment options, including psychotherapy, psychotropic medication, relaxation techniques, natural remedies, acupuncture and meditation. By means of a 5-point Likert scale ranging from “would strongly recommend” to “would not recommend at all” respondents could express their treatment preferences.

### 2.4 Statistical Analysis

We carried out analyses on item level for the combined sample, using multinomial logit regression models and collapsing the five-point Likert scales into three categories (*e.g.*, “agree”, “undecided”, “disagree”). These models yield predicted probabilities for choosing each category in Germany and Tunisia as well as the predicted differences (probability change) between both countries, all given as percentages. In addition, multinomial logit regressions were used for comparing beliefs about schizophrenia and depression within each country.

## 3 RESULTS

### 3.1 Comparison of Public Beliefs about the Causes of Schizophrenia and Depression

As reported in Table **2** (for raw percentages see Table **A1** in APPENDIX A), the probability that respondents endorsed factors representing current stress as cause of schizophrenia was significantly higher in Tunisia than in Germany. Except for sexual abuse, the same held true for causes subsumed under childhood adversities. By contrast, in Tunisia the majority ofrespondents disapproved of biogenetic explanations while in Germany the majority was in favor of them. With depression, a somewhat similar pattern emerged. Again, the inclination to agree with psychosocial explanations was generally higher in Tunisia, only stressful life events and work-related stress were equally frequently endorsed in both countries. As with schizo-phrenia, in Tunisia biogenic factors were considered less frequently as a cause than in Germany, although the difference between both countries was smaller. Differences between both disorders in the endorsement of biogenetic causes and current stress (exception stressful life events) were more pronounced in Germany than in Tunisia (Table **B1** in APPENDIX B).

Across both disorders, respondents in Tunisia were markedly more likely than in Germany to hold the person afflicted responsible for his or her illness. In Tunisia, there was also a strong tendency to perceive both disorders as punishment for not having faith in God (schizophrenia 61%, depression 59%) and failing in religious commitment (schizophrenia 54%, depression 45%). Less frequently mental disorders were attributed to the possession by djinns (schizophrenia 22%, depression 12%).

### 3.2 Comparison of Help-seeking Recommendations for Schizophrenia and Depression

As reported in Table **3** (for raw percentages see Table **A2** in APPENDIX A), mental health professionals were strongly advocated in both countries, for the treatment of depression in Tunisia even more than in Germany. By contrast, GPs were endorsed in Tunisia across both disorders markedly less frequently than in Germany. In Tunisia, applying for a health cure was less popular than in Germany. The largest difference between both countries was observed as concerns religious help. The involvement of an imam was recommended in Tunisia over four-times as frequently than seeing a priest in Germany. Turning to an exorcist/traditional healer was considered an option in Tunisia by only a small minority (8%). Consulting the Internet was not very popular in both countries, in Tunisia even less than in Germany. Talking to a confidant was appreciated as a source of help by the majority of respondents, in case of schizophrenia slightly more in Tunisia than in Germany, in case of depression more in Germany than in Tunisia.

### 3.3 Comparison of Treatment Recommendations for Schizophrenia and Depression

The treatment preferences of the public are reported in Table **4** (for raw percentages see Table **A3** in APPENDIX A). While, irrespective of the disorder, psychotherapy was the clear favorite in both countries, the readiness to endorse it was significantly higher in Tunisia. Relaxation techniques and meditation were also highly recommended in Tunisia, markedly more than in Germany. By contrast, in Tunisia, there was a strong opposition to medication while in Germany over half recommended medication for the treatment of schizophrenia and there was some ambivalence about the treatment of depression. Across both disorders, natural remedies were advised against twice as frequently in Tunisia than in Germany. As concerns acupuncture the picture was mixed: respondents from Tunisia were more opposed in case of schizophrenia and more in favor in case of depression than those from Germany. Differences between both disorders in the endorsement of established forms of treatment were more pronounced in Germany than in Tunisia (Table **B3** in APPENDIX B).

## 4 DISCUSSION

### 4.1 Differences in Public Beliefs about Mental Disorders between Tunisia and Germany

Over the last decades, the public in Germany as in other western countries has been increasingly exposed to information on biochemical and genetic etiological theories, with the consequence that a bio-medical model of mental disorders nowadays enjoys growing popularity [2]. This may be one of the reasons why in Germany over twice as many respondents endorsed disturbances in the brain or genetic factors as a cause than in Tunisia, where respondents were more inclined to endorse psychosocial causes like current stress or childhood adversities. Instead of conceptualizing the deviant behavior described in the vignette as originating from a disease located in the individual, Tunisian people were more inclined to seek the cause for the condition in difficulties of life. Against this backdrop, it is no surprise that in Tunisia psychotherapy, as well as, relaxation techniques and meditation were more frequently recommended than in Germany, while medication, but also natural remedies (a ‘softer’ form of biological treatment), were met with more reservation.

While in Germany, a largely secularized western country, medical-psychiatric conceptualizations of mental illness have permeated the public notion of mental illness, in Tunisia, a country with around 98% of the population being Muslims, religious beliefs are of great importance. Traditionally, Islam views mental disorders as an outcome of an inadequate relationship with God. According to this perception, an inadequate relationship with God can be expressed by the failure to observe the laws of Islam as prescribed in the Koran and the petitions of the Prophet Muhammad [19]. In fact, over half of our Tunisian respondents attributed the mental disorders described in the vignettes to a lack of faith in Allah or to insufficient religious commitment. By contrast, in a survey conducted in Germany some years ago only a few persons saw in the occurrence of schizophrenia or depression the expression of God’s will (7% in the West and 3% in the East) [20]. Correspondingly, a considerable proportion of our Tunisian respondents embraced the endorsement of the help offered by an imam who may help remedy disconnections from God. In Germany, by contrast, only a tiny minority recommended turning to a priest, which among the seven options offered was the one that was least frequently chosen. In view of the steadily growing influence of a more conservative Islam in Arab countries, it appears not unlikely that the gap between both countries may even increase in the near future.

According to our findings, the belief that mental illness may be due to the possession by evil spirits (djinns) seems relatively rarely shared by the general public and only a few recommended visiting a traditional healer or an exorcist. However, studies conducted at the psychiatric hospital Razi in Tunis suggest that magic beliefs may be in fact more prevalent. Bouhlel *et al*. (2013) [21] report that among relatives of patients with schizophrenia magic explanations were approved of by almost half and over half recommended traditional treatments like exorcism. Similarly, in another study, almost two-thirds of mothers attributed the first psychotic episode of their child to spirit possession [22]. In fact, half of the patients admitted to ward of the same hospital reported having already tried traditional therapy [23].

In addition to prevailing religious values also the scarcity of available mental health services may have contributed to the popularity of religious advisors [24]. While in Germany one psychiatrist does serve 6,000 people [26], in Tunisia the ratio amounts to 1 to 33,000. The difference in density of psychotherapists is even more pronounced: 1 to 2,500 [25] versus 1 to 23,000. Against this backdrop, it appears somewhat paradoxical that almost all respondents in Tunisia advocated seeking help from a mental health professional, as concerns depression even more frequently than their counterparts in Germany.

Tunisians tended much more than Germans to share moralistic views and to blame the afflicted person for his or her illness. As we have already learned, the opposite was found with biogenetic explanations which were markedly more frequently endorsed by Germans than by Tunisians. The discrepancy is particularly pronounced with schizophrenia: While, for instance, in Tunisia 77% attributed the onset of the illness to a weakness of will and only 20% to a brain disease, in Germany the respective percentages were 28% and 63%. This finding is quite intriguing and one may infer from it that there is a reciprocal relationship between biological and moralistic attributions. In consequence, one might expect that by promulgating the biomedical model one may succeed in avoiding people being blamed for their illness – and help reduce the stigma attached to schizophrenia. Unfortunately, studies from Germany as well as from other countries do not support this hope. With the increasing popularity of biogenetic conceptualizations of schizophrenia the public’s desire for social distance from those afflicted has not decreased, rather the opposite is the case [2, 26].

In Germany, a clear line between schizophrenia and depression was drawn whereas in Tunisia a more uniform notion of mental illness seemed to prevail. It is tempting to consider this as an indication of greater ‘mental health literacy’ of the German public, which may, at least in part, have resulted from numerous efforts to raise the public’s awareness and to increase its knowledge of mental disorders that have been made in recent years [27]. In fact, findings such as that in Germany biogenic causes were significantly more frequently held responsible for schizophrenia than for depression and the opposite was found for current stress, support this view. Also, the finding that for the treatment of schizophrenia medication was more recommended and ‘alternative’ methods (relaxation, meditation) less, point in this direction. But what about the finding that Germans, in contrast to Tunisians, recommended psychotherapy more frequently for the treatment of schizophrenia than for the treatment of depression, which is certainly not in agreement with official guidelines? A possible explanation for this rather unexpected result may be that the lay public in Germany did not necessarily understand by the term psychotherapy the same as professionals. People may have thought more of ‘talk therapy’ or simply ‘talking with the person’ rather than of the application of specific psychotherapeutic techniques [28]. Another finding that raises some doubts about the German public’s superior mental health literacy is that respondents from Tunisia advised more frequently than those from Germany against the use of natural remedies.

While illness beliefs of the Tunisian public hardly showed any gender differences they varied significantly according to age: Younger respondents endorsed less frequently psychosocial stress as a cause of mental disorder; they also attributed mental disorder less frequently to insufficient faith in Allah and, consequently, recommended less turning to an Imam or an exorcist. Instead, younger respondents were more ready recommending seeking help from mental health professionals or consulting the Internet. However, they also were more in favor of using relaxation techniques and ‘alternative’ treatments. Apart from reflecting changes in beliefs over the life span, age differences may represent so-called cohort effects [29] due to younger generations being more exposed to western conceptualizations of mental illness and modern trends in health care preferences. Thus, the differences observed between Tunisia and Germany may become less pronounced over time.

### 4.2 Strengths and Limitations

The strength of our study is that it is based on the first vignette-based population-based survey on attitudes and beliefs about mental illness that has been conducted in North Africa on a national scale. The fact that in both countries the same interview has been used for measuring attitudes and beliefs represents another strength of this study. However, although great pains have been taken with the translation of the interview, linguistic equivalence does not necessarily mean cultural equivalence. For methodological reasons, illiterate persons had been excluded from the Tunisian sample. As traditional beliefs about mental illness may prevail among them more than among better educated people differences between both countries may be even more pronounced than those observed in this study.

## CONCLUSION

Marked differences between Tunisia and Germany exist in public beliefs about the causes of mental disorders and their treatment, which correspond to differences in cultural orientations prevailing in these countries. Mental health professionals need to be sensitive to the particular cultural context in which they operate, in order to be able to reach those they intend to care for. In Tunisia, this may include tolerating views and practices that are alternative to the tenets of western psychiatry and trying to integrate them in the treatment of patients [23, 30].

## Figures and Tables

**Table 1:** Socio-demographic characteristics of both samples.

Tunisia			Germany		
-	Sample %	General population (15-64 years)^1^ %	-	Sample %	General population (15+ years)^2^ %
Sex Male Female	51.8 48.2	49.3 50.7	Sex Male Female	45.6 54.4	48.6 51.4
Age (years) 15-29 30-44 45-64	41.2 30.9 27.9	40.1 32.0 27.9	Age 18-25 26-45 46-60 61+	8.5 30.7 28.5 32.3	11.3 31.9 26.9 29.9
Marital status Married Single Other	46.0 53.5 0.5	41.2 54.1 4.7	Marital status Married Single Other	54.6 28.3 17.1	52.9 30.6 16.5
Educational attainment Illiterate/less than primary school Primary school Secondary school and more	5.1 46.2 48.6	25.9 31.6 36.9	Educational attainment Still student No schooling complited 8/9 years of schooling 10 years of schooling and more	0.0 3.4 39.6 57.1	1.0 4.0 38.5 56.4

^1^ Data from the Statistical Office of Tunisia for 2013.

^2^ Data from the Federal Statistical Office of Germany for 2011.

**Table 2:** Beliefs about the causes of schizophrenia and depression: Comparison between Tunisia and Germany (multinomial logit regressions).

-		Schizophrenia			Depression		
		Predicted Percentages			Predicted Percentages		
		Tunisia	Germany	Difference [95% CI]	Tunisia	Germany	Difference [95%CI]
Brain disease	Agree Undecided Disagree	**20** **9** **70**	**63** **19** **18**	**-42 [-49, -36]** **-10 [-15, -5]** **52 [46, 59** **]**	**13** **5** **82**	**30** **28** **42**	**-16 [-22, -11]** **-24 [-0.28, -19]** **40 [34, 46** **]**
Chemical imbalance in the brain	Agree Undecided Disagree	**23** 12 **65**	**57** 26 **17**	**-34 [-41, -27]** -14 [-20, -8] **48 [ 41, 55**]	**15** **9** **76**	**34** **30** **36**	**-20 [-26, -14]** **-21 [-26, -15]** **40 [33, 47** **]**
Heredity	Agree Undecided Disagree	**21** 12 **67**	**45** 25 30	**-24 [-31, -17]** -13 [-19, -8] **37 [****30****, 44]**	**17** **11** **72**	**30** **27** **42**	**-13 [-20, -7]** **-17 [-22, -11]** **30 [** **23** **, 37** **]**
Stressful life event	Agree Undecided Disagree	**77** 10 12	**69** 21 11	**8 [****2****, ****15****]** -11 [-16, -6] 2 [-3, 7]	78 **10** 12	75 **16** 9	3 [-3, 10] **-6 [-11, -1]** 3 [-2, 8]
Troubles in partnership/family	Agree Undecided Disagree	**77** **12** **11**	**55** **27** **18**	**22 [** **15** **, ** **29** **]** **-15 [-20, -9]** **-7 [-12, -2]**	**81** **10** 9	**74** **18** 9	**8 [****2****, ****14****]** **-8 [-13, -3]** 0 [-4, 4]
Work-related stress	Agree Undecided Disagree	76 10 14	**62** **22** 16	**15 [****7****, ****21****]** **-12 [-17, -7]** -2 [-7, 3]	81 **5** **13**	80 **14** **6**	2 [-4, 7] **-9 [-12, -5]** **7 [****2****, ****11****]**
Lack of parental affection	Agree Undecided Disagree	**55** **10** 36	**34** **28** 38	**20 [****13****, ****28****]** **-18 [-23, -13]** -2 [-9, 5]	**67** **9** **24**	**32** **30** **39**	**35 [** **28** **, 42** **]** **-21 [-26, -16]** **-15 [-21, -8]**
Sexual abuse	Agree Undecided Disagree	34 **16** **50**	31 **27** **42**	3 [-4, 10] **-11 [-07, 5]** **8 [ 0, 16]**	**42** **11** 47	**22** **28** 50	**20 [****12****, ****27****]** **-18 [-23, -12]** -2 [-10, 6]
Broken home	Agree Undecided Disagree	**77** **12** **11**	**55** **27** **18**	**22 [** **15** **, ** **29** **]** **-15 [-0.20, -9]** **-7 [-12, -2]**	**81** **10** 9	**74** **17** 9	**8 [****2****, ****14****]** **-8 [-13, 3]** 0 [-4, 4]
Immoral life style	Agree Undecided Disagree	**31** **12** 57	**18** **19** 62	**12 [****6****, ****19****]** **-8 [-13, -2]** -5 [-12, 3]	**39** 15 **46**	**16** 20 **64**	**24 [****16****, ****30****]** -5 [-11, 1] **-18 [-26, -10]**
Weak will	Agree Undecided Disagree	**77** **10** **13**	**28** **23** **50**	**49 [41, 58** **]** **-13 [-20, -6]** **-37 [-45, -29]**	**74** **8** **18**	**22** **26** **52**	**52 [44, 60** **]** **-18 [-25, -10]** **-34 [-43, -25]**

Statistically significant differences in bold.

**Table 3:** Help-seeking recommendations for schizophrenia and depression: Comparison between Tunisia and Germany (multinomial logit regressions).

-		Schizophrenia			Depression		
		Predicted Percentages			Predicted Percentages		
		Tunisia	Germany	Difference [95% CI]	Tunisia	Germany	Difference [95% CI]
Confidant	Recommend Undecided Advise against Don´t know	**83** **8** 9 0	**74** **16** 8 2	**9 [****3****, ****15****]** **-8 [-13, -4]** 1 [-3, 5] -1 [-3, 0]	73 12 **14** 1	80 13 **5** 2	-7 [-14, 0] -0 [-6, 5] **9 [****4****, ****14****]** -1 [-3, 0]
Imam/Priest	Recommend Undecided Advise against Don´t know	**72** **12** **15** **1**	**16** **22** **49** **14**	**56 [49, 62]** **-10 [-15, -4]** **-33 [-39, -27]** **-13 [-16, -10]**	**64** **9** **24** **2**	**15** **19** **54** **11**	**49 [42, 56** **]** **-10 [-15, -5]** **-30 [-37, -23]** **-9 [-12, -6]**
Internet	Recommend Undecided Advise against Don´t know	**9** **7** **82** **2**	**18** **23** **49** **10**	**-9 [-13, -4]** **-16 [-21, -11]** **33 [** **27** **, 39** **]** **-8 [-11, -5]**	16 **8** **74** **2**	20 **27** **43** **10**	-4 [-0., 1] **-18 [-24, -13]** **31 [****24****, 40]** **-9 [-12, -6]**
Health cure	Recommend Undecided Advise against Don´t know	35 **12** **51** **2**	40 **26** **25** **9**	-6 [-13, 2] **-13 [-19, -8]** **26 [****19****, 33]** **-7 [-9, -4]**	**44** **15** **37** 4	**55** **22** **17** 6	**-11 [-19, -4]** **-7 [-12, -1]** **20 [****13****, ****26****]** -2 [-5, 1]
G.P.	Recommend Undecided Advise against Don´t know	**49** 11 **40** 0	**74** 13 **13** 0	**-25 [-33, -18]** -2 [-8, 3] **27 [****20****, 34]** 0 [0, 1]	27 14 **59** 0	**77** 14 **9** 0	**-50 [-57, -44]** -0 [-6, 5] **51 [44, 57]** 0 [0, 1]
Psychotherapist	Recommend Undecided Advise against Don´t know	86 6 7 1	88 7 3 2	-1 [-6, 4] -1 [-5, 2] 4 [ 0, 7] -1 [-2, 0]	**90** **3** 6 1	76 **12** 9 2	**14 [ 08, 19]** **-10 [-13, -6]** -3 [-7, 1] -2 [-3, 0]
Psychiatrist	Recommend Undecided Advise against Don´t know	**90** **3** 6 1	**82** **10** 6 2	**7 [****3****, ****12****]** **-6 [-10, -3]** 0 [-3, 4] -1 [-3, 0]	89 3 8 0	68 15 15 2	22 [16, 27] -13 [-17, -9] -7 [-11, -2] -2 [-3, -1]

Statistically significant differences in bold

**Table 4:** Treatment recommendations for schizophrenia and depression: Comparison between Tunisia and Germany (multinomial logit regressions).

-		Schizophrenia			Depression		
		Predicted Percentages			Predicted Percentages		
		Tunisia	Germany	Difference [95% CI]	Tunisia	Germany	Difference [95% CI]
Psychotherapy	Recommend Undecided Advise against Don´t know	**91** **2** 6 **1**	**84** **8** 4 **5**	**7 [****3****, ****12****]** **-6 [-8, -3]** 2 [-7, 6] **-4 [-6, -2]**	**91** **2** **6** **0**	**74** **10** **10** **6**	**18 [** **12** **, ** **23** **]** **-8 [-11, -5]** **-4 [-8, -1]** **-6 [-8, -4]**
Psychotropic medication	Recommend Undecided Advise against Don´t know	**29** **11** **57** **2**	**51** **23** **17** **10**	**-22 [-29, -15]** **-11 [-16, -6]** **40 [33, 47** **]** **-7 [-10, -4]**	37 **10** **50** **2**	36 **21** **33** **10**	2 [-6, 9] **-11 [-16, -6]** **17 [ 09, 25]** **-7 [-11, -4]**
Relaxation techniques	Recommend Undecided Advise against Don´t know	**72** **12** **14** **2**	**43** **26** **23** **9**	**30 [** **23** **, 37** **]** **-15 [-20, -9]** **-8 [-14, -3]** **-7 [-10, -4]**	**72** **12** **9** 7	**53** **25** **15** 7	**19 [****12****, ****26****]** **-13 [-18, -7]** **-6 [-11, -2]** 0 [-4, 4]
Natural remedies	Recommend Undecided Advise against Don´t know	**11** **11** **76** **2**	**24** **26** **37** **13**	**-13 [-18, -8]** **-15 [-20, -9]** **40 [ 32, 46** **]** **-11 [-14, -8]**	**20** **10** **69** **0**	**27** **30** **31** **11**	**-7 [-14, -1]** **-20 [-25, -14]** **38 [31, 45** **]** **-11 [-14, -8]**
Acupuncture	Recommend Undecided Advise against Don´t know	16 **8** **62** 14	17 **23** **42** 18	-1 [-6, 5] **-15 [-20, -11]** **20 [****12****, ****27****]** -4 [-10, 2]	**28** **11** 42 19	**18** **24** 39 19	**10 [****4****, ****17****]** **-13 [-18, -8]** 3 [-4, 11] -1 [-7, 6]
Meditation	Recommend Undecided Advise against Don´t know	**51** **11** 30 9	**31** **30** 26 13	20 [12, 27] -19 [25, -14] 4 [-3, 11] -4 [-9, 0]	**52** **9** 24 14	**41** **28** 20 11	**11 [****3****, ****19****]** **-18 [-24, -13]** 4 [-2, 11] 3 [-2, 9]

Statistically significant differences in bold.

## APPENDIX A

**Table A1:** Beliefs about the causes of schizophrenia and depression: Comparison between Tunisia and Germany (raw percentages).

		Schizophrenia		Depression		
		Tunisia	Germany	Tunisia		Germany
Brain disease	Agree Undecided Disagree	21 10 69	62 19 19	14 5 80		29 28 43
Chemical imbalance in the brain	Agree Undecided Disagree	24 13 64	57 26 18	15 10 75		34 30 36
Heredity	Agree Undecided Disagree	22 12 66	44 25 31	18 11 71		29 28 43
Stressful life event	Agree Undecided Disagree	80 9 11	66 23 11	81 8 11		73 17 10
Troubles in partnership/family	Agree Undecided Disagree	77 11 12	54 28 18	81 9 10		73 18 9
Work-related stress	Agree Undecided Disagree	75 10 14	62 22 16	81 5 14		79 14 7
Lack of parental affection	Agree Undecided Disagree	55 10 36	34 28 38	67 9 24		32 30 39
Sexual abuse	Agree Undecided Disagree	40 16 44	29 27 44	50 11 40		21 27 52
Broken home	Agree Undecided Disagree	77 12 11	55 27 18	81 10 9		74 17 9
Immoral life style	Agree Undecided Disagree	35 11 54	17 20 63	44 14 42		15 20 64
Weak will	Agree Undecided Disagree	78 10 12	26 23 51	75 8 17		22 25 53

**Table A2:** Help-seeking recommendations for schizophrenia and depression: Comparison between Tunisia and Germany (raw percentages).

		Schizophrenia		Depression		
		Tunisia	Germany		Tunisia	Germany
Confidant	Recommend Undecided Advise against Don´t know	83 8 8 2	73 17 8 2		74 12 13 1	79 13 5 2
Imam/Priest	Recommend Undecided Advise against Don´t know	75 11 13 1	15 22 51 12		68 8 21 3	14 19 57 10
Internet	Recommend Undecided Advise against Don´t know	10 7 79 3	18 23 49 10		18 8 71 3	20 27 44 10
Health cure	Recommend Undecided Advise against Don´t know	33 12 51 3	41 26 25 8		42 15 37 6	55 22 17 5
G.P.	Recommend Undecided Advise against Don´t know	47 10 42 1	73 13 12 2		25 13 61 1	76 14 9 1
Psychotherapist	Recommend Undecided Advise against Don´t know	87 5 7 1	86 9 3 2		90 2 7 1	75 14 9 2
Psychiatrist	Recommend Undecided Advise against Don´t know	90 3 6 1	82 11 6 2		89 2 8 0	67 16 15 2

**Table A3:** Treatment recommendations for schizophrenia and depression: Comparison between Tunisia and Germany (raw percentages).

		Schizophrenia		Depression	
		Tunisia	Germany	Tunisia	Germany
Psychotherapy	Recommend Undecided Advise against Don´t know	92 2 6 1	84 8 4 4	92 2 6 0	73 11 11 6
Psychotropic medication	Recommend Undecided Advise against Don´t know	28 12 56 3	51 22 17 10	36 11 50 3	36 21 33 10
Relaxation techniques	Recommend Undecided Advise against Don´t know	71 13 14 2	43 26 23 9	70 14 9 8	53 24 16 7
Natural remedies	Recommend Undecided Advise against Don´t know	13 14 71 2	24 25 39 12	24 12 64 0	27 29 34 10
Acupuncture	Recommend Undecided Advise against Don´t know	17 7 59 17	17 23 43 17	29 10 40 18	18 24 40 18
Meditation	Recommend Undecided Advise against Don´t know	47 10 27 15	32 30 27 12	48 9 22 22	42 27 21 10

## APPENDIX B

**Table B1:** Beliefs about the causes of mental disorders: Comparison between depression and schizophrenia (multinomial logit regressions).

		Tunisia			Germany		
		Predicted Percentages			Predicted Percentages		
		Depression	Schizophrenia	Difference [95%CI]	Depression	Schizophrenia	Difference [95%CI]
Brain disease	Agree Undecided Disagree	**13** **5** **82**	**20** **9** **70**	**-7 [-12, -2]** **-4 [-8, -1]** **11 [** **6** **, ** **17** **]**	**30** **28** **42**	**63** **19** **18**	**-33 [-37, -28]** **9 [** **5** **, ** **13** **]** **23 [** **19** **, ** **28** **]**
Chemical imbalance in the brain	Agree Undecided Disagree	**15** 9 **76**	**23** 12 **65**	**-9 [-14, -3]** -3 [-7, 2] **11 [****5****, ****18****]**	**34** 30 **36**	**57** 26 **17**	**-23 [-27, -18]** 4 [0, 8] **19 [****15****, ****23****]**
Heredity	Agree Undecided Disagree	17 11 72	21 12 67	-4 [-10, 1] -1 [-5, 4] 5 [-1, 12]	**30** 27 **42**	**45** 25 **30**	**-15 [-18, -9]** 3 [-2, 7] **12 [****8****, ****17****]**
Stressful life event	Agree Undecided Disagree	78 10 12	77 10 12	1 [-6, 7] -1 [-5, 4] 0 [-5, 5]	**75** **16** 9	**69** **21** 11	**6 [****2****, ****10****]** **-5 [-9, -16]** -1 [-4, 2]
Troubles in partnership/family	Agree Undecided Disagree	81 10 9	77 12 11	5 [-1,10] -2 [-7, 2] -2 [-6, 2]	**74** **18** **9**	**55** **27** **18**	**19 [** **14** **, ** **23** **]** **-9 [-13, -5]** **-10 [-13, -6]**
Work-related stress	Agree Undecided Disagree	81 **5** 13	76 **10** 14	6 [0, 11] **-5 [-9, -1]** -1 [-5, 4]	**80** **14** **6**	**62** **22** **16**	**18 [** **14** **, ** **22** **]** **-18 [-12, -5]** **-10 [-13, -7]**
Lack of parental affection	Agree Undecided Disagree	**67** 9 **24**	**55** 10 **36**	**12 [****5****, ****19****]** -1 [-1, 3] **-12 [-18, -5]**	32 30 39	34 28 38	-3 [-7, 2] 2 [-3, 6] 1 [-3, 6]
Sexual abuse	Agree Undecided Disagree	**42** 11 47	**34** 16 50	**8 [****1****, ****15****]** -5 [-10, 0] -3 [-10, 5]	**22** 28 **50**	**31** 27 **42**	**-9 [-13, -5]** 1 [-3, 5] **7 [****3****, ****12****]**
Broken home	Agree Undecided Disagree	81 10 9	77 12 11	5 [-1, 10] -2 [-7, 2] -2 [-6, 2]	**74** **17** **9**	**55** **27** **18**	**19 [** **14** **, ** **23** **]** **-9 [-13,-5]** **-10 [-13, -6]**
Immoral life style	Agree Undecided Disagree	**39** 15 **46**	**31** 12 **57**	**8 [****1****, ****15****]** 3 [-2, 8] **-12 [-19, -5]**	16 20 64	18 19 62	-2 [-6, 1] 1 [-3, 5] 1 [-3, 6]
Weak will	Agree Undecided Disagree	74 8 18	77 10 13	-3 [-9, 3] -2 [-6, 2] 4 [-1, 10]	22 26 52	28 23 50	-5 [-11, 1] 3 [-3, 9] 2 [-4, 9]

Statistically significant differences in bold.

**Table B2:** Help-seeking recommendations for mental disorders: Comparison between depression and schizophrenia (multinomial logit regressions).

		Tunisia			Germany		
		Predicted Percentages			Predicted Percentages		
		Depression	Schizophrenia	Difference [95% CI]	Depression	Schizophrenia	Difference [95% CI]
Confidant	Recommend Undecided Advise against Don´t know	**73** 12 14 1	**83** 8 9 0	**-10 [-15, -4]** 4 [0, 9] 5 [0, 10] 0 [-1, 1]	**80** 13 **5** 2	**74** 16 **8** 2	**6 [****2****, ****10****]** -3 [-7, 0] **-3 [-5, -1]** 0 [-1, 2]
Imam/Priest	Recommend Undecided Advise against Don´t know	**64** 9 **24** 2	**72** 12 **15** 1	**-7 [-14, -1]** -3, [-7, 15] **9 [****3****, ****15****]** 2 [0, 3]	15 19 **54** 11	16 22 **49** 14	-1 [-4, 3] -3 [-7, 1] 5 [1, 10] -2 [-5, 1]
Internet	Recommend Undecided Advise against Don´t know	**16** 8 **74** 2	**9** 7 **82** 2	**6 [****2****, ****11****]** 1 [-3, 5] **-8 [-13, -2]** 0 [-1, 1]	20 27 **43** 10	18 23 **49** 10	2 [-2, 6] 3 [-1, 7] **-6 [-10, -1]** 0 [-3, 3]
Health cure	Recommend Undecided Advise against Don´t know	**44** 15 **37** 4	**35** 12 **51** 2	9 [2, 16] 3 [-2,8] **-14 [-21,-7]** 2 [0,4]]	**55** 22 **17** 6	**40** 26 **25** 9	**14 [****10****, ****19****]** -4 [-8, 0] **-8 [-12, -4]** -3 [-5, 0]]
G.P.	Recommend Undecided Advise against Don´t know	**27** 14 **59** 0	**49** 11 **40** 0	**-22 [-29, -16]** 3 [-2, 8] 19 [12, 26] 0 [-1, 0]	77 14 **9** 0	74 13 **13** 0	3 [-1, 7] 1 [-2, 4] -4 [-7, -1] 0
Psychotherapist	Recommend Undecided Advise against Don´t know	90 3 6 1	86 6 7 1	4 [-1, 8] -3 [-6, 5] -1 [-4, 3] 0 [-1, 1]	**76** **12** **9** 2	**88** **7** **3** 2	**-11 [-15, -8]** **5 [****2****, ****8****]** **6 [****4****, ****8****]** 0 [-1, 2]
Psychiatrist	Recommend Undecided Advise against Don´t know	89 3 8 0	90 3 6 1	-1 [-5, 4] -1 [-3, 2] 2 [-2, 5] 0 [-1, 0]	**68** **15** **15** 2	**82** **10** **6** 2	**-15 [-19, -11]** **6 [****3****, ****9****]** **9 [****6****, ****12****]** 0 [-1, 2]

Statistically significant differences in bold.

**Table B3:** Treatment recommendations for mental disorders: Comparison between depression and schizophrenia (multinomial logit regressions).

		Tunisia			Germany		
		Predicted Percentages			Predicted Percentages		
		Depression	Schizophrenia	Difference [95% CI]	Depression	Schizophrenia	Difference [95% CI]
Psychotherapy	Recommend Undecided Advise against Don´t know	91 2 6 0	91 2 6 1	0 [-4, 4] 1 [-2, 3] 0 [-3, 3] 0 [-1, 0]	**74** 10 **10** 6	**84** 8 **4** 5	**-10 [-14, -7]** 3 [0, 5] **6 [****4****, ****9****]** 1 [-1, 3]
Psychotropic medication	Recommend Undecided Advise against Don´t know	**37** 10 50 2	**29** 11 57 2	**8 [****2****, ****15****]** -1 [-6, 3] -7 [-13, 0] 0 [-2, 2]	**36** 21 **33** 10	**51** 23 **17** 10	**-15 [-20, -11]** -2 [-5, 2] **17 [****13****, ****21****]** 0 [-3, 3]
Relaxation techniques	Recommend Undecided Advise against Don´t know	72 12 9 **7**	72 12 14 **2**	0 [-7, 6] 1 [-4, 5] -5 [-10, 0] **5 [****2****, ****8****]**	**53** 25 **15** 7	**43** 26 **23** 9	**10 [****6****, ****15****]** -1 [-5, 3] **-7 [-11, -4]** -2 [-4, 1]
Natural remedies	Recommend Undecided Advise against Don´t know	**20** 10 **69** 0	**11** 11 **76** 2	**9 [****4****, ****14****]** -1 [-5, 3] **-7 [-13, -1]** -1 [-3, 0]	27 30 **31** 11	24 26 **37** 13	3 [-1, 7] 4 [0, 8] **-6 [-10, -2]** -1 [-5,2]
Acupuncture	Recommend Undecided Advise against Don´t know	**28** 11 **42** 19	**16** 8 **62** 14	**12 [****6****,****18**] 3 [-1, 7] **-20 [-26, -13]** 4 [-1, 9]	18 24 39 19	17 23 42 18	1 [-2, 5] 1 [-3, 5] -3 [-8,1] 1 [-3, 5]
Meditation	Recommend Undecided Advise against Don´t know	52 9 24 14	51 11 30 **9**	2 [-5, 9] -1 [-6, 3] -5 [-12, 1] **5 [****1****, ****9****]**	**41** 28 **20** 11	31 30 **26** 13	**10 [****6****, ****17****]** -2 [-6, 2] **-6 [-10, -2]** -2 [-5, 1]

Statistically significant differences in bold.
